# Determinants of household vulnerability to food insecurity during COVID-19 lockdown in a mid-term period in Iran

**DOI:** 10.1017/S1368980021000318

**Published:** 2021-05

**Authors:** Mohammad Reza Pakravan-Charvadeh, Moselm Savari, Haider A Khan, Saeid Gholamrezai, Cornelia Flora

**Affiliations:** 1 Department of Agricultural Economics and Rural Development, Faculty of Agriculture, Lorestan University, Khorramabad, Lorestan, Iran; 2 Department of Agricultural Extension and Education, Agricultural Sciences and Natural Resources, University of Khuzestan, Mollasani, Iran; 3 Department of Economics and Josef Korbel School of International Studies, University of Denver, Denver, USA; 4 Department of Sociology, Iowa State University, Iowa, USA

**Keywords:** COVID-19 lockdown, household food insecurity, public health, vulnerability, Iran

## Abstract

**Objective:**

This study aimed to identify and rank the different aspects of households’ vulnerability to food insecurity.

**Design:**

The data were collected by a standard online questionnaire. The Household Food Insecurity Access Scale was used to assess food insecurity levels, and first-order structural equation modelling was applied to determine factors that affect food insecurity. Seven dimensions of vulnerability were measured: economic, social, cultural, human, physical, psychology and information, using thirty-seven items extracted from the related literature review.

**Setting:**

This study was implemented in Tehran province in Iran.

**Participants:**

The sample included 392 families residing in Tehran province which was determined using random sampling.

**Results:**

About 61 % of the total sample faced food insecurity, at marginal, moderate and severe levels. Economic, psychological and human aspects of vulnerability had the highest effect on food insecurity during the initial COVID-19 lockdown.

**Conclusions:**

Authorities and policymakers must provide economic and financial support to vulnerable households. Abolition of US economic and financial sanctions imposed on Iran must be implemented to battle with COVID-19 in this country.

The COVID-19 outbreak brought severe challenges for people around the world, including in health and nutrition^(1)^. This disease began with animal-to-human infection in Wuhan, Hubei, China in late 2019^(2)^, causing growing concerns about the quality of life globally. Given the growing outbreak of COVID-19 cases and deaths around the world^(3)^, the fears of household vulnerability to food insecurity are increasing globally^(4)^. Vulnerability is the risk to livelihood at a specific time in the future affecting the level of household or individual welfare and quality of life, and finally food security level^(5)^.

Food security exists ‘when all people, at all times, have physical and economic access to sufficient, safe and nutritious foods meeting their dietary needs and food preferences for an active and healthy life’^(6)^. About 212 countries and territories are affected by COVID-19 around the world. Iran as other countries is seriously affected by this pandemic disease. Official reports demonstrate that more than 1 206 373 people were infected and almost 54 814 lost their lives to COVID-19 until January 1. To reduce the spread of the virus, the Iranian government executed several coping strategies, including quarantine, isolation and social distancing. Unlike the previous studies which assessed the vulnerability of households in different countries in a stable and non-viral environment^(7–10)^, the present study assesses the vulnerability of households to food insecurity during the COVID-19 outbreak.

Vulnerability is the level to which a human population is sensitive to the effect of a threat and a catastrophe arises when a susceptible population is exposed by the impact of a hazard^(10,11)^. Vulnerability is the conditions determined by economic, social, physical and environmental factors or processes, which augment the susceptibility of a community to the effect of threats^(12,13)^. Vulnerability assessment can be classified into two distinct categories: (a) models analysing vulnerability to stochastic events, including shocks, hazards or risks, usually in a short term – and (b) models analysing vulnerability to the outcomes of those shocks which is used for a long-term assessment^(14–16)^.

It seems that global scrambles to decrease COVID-19 jeopardy are gradually being viewed within the context of sustainable development^(17)^. Due to the short-term period of COVID-19 pandemic disease, a stochastic event is considered as vulnerability dimension^(18)^. Some contend that vulnerability should be assessed by considering several aspects including social, economic, environmental and human capital^(19)^. Other identified natural and physical capitals as important factors for measuring vulnerability to promote disaster-resilient societies^(5,10,11,20–22)^.

The results of the present study will help policymakers and public health authorities identify the different factors affecting households’ vulnerability during the next phase of the COVID-19 outbreak or a virus in the future. The results can also be used to cope with households’ vulnerability during the COVID-19 outbreak, emphasising different capitals, including social, economic, human, physical and psychological aspects.

Considering the urgent need to assess vulnerability of household’s sustainable livelihood to food insecurity during COVID-19 outbreak, this study will:

- assess the status of food insecurity of Iranian households in a mid-term period.
- determine the effect of household vulnerability and sustainable livelihood to food insecurity.
- rank the different categorisations of households’ capital on vulnerability to food insecurity.
- rank the different actions to reduce the vulnerability to food insecurity in a mid-term period.

## Materials and methods

### Cross-sectional framework

The present study is quantitative and practical research. It is a cross-sectional research (correlational type), and a structural model was used to follow the goals of the study. The questionnaire was designed based on the literature and opinions of the experts, and then the data were collected through an online survey^(18)^. Food insecurity status of the households was determined during COVID-19 outbreak. Finally, the correlation of different aspects of vulnerability of households’ sustainable livelihood to food insecurity during COVID-19 pandemic disease was determined.

### Study area and population

An online questionnaire was used to collect data during 1 April to 31 July in Tehran province, the first-largest province in Iran, and second-largest metropolitan area in the Middle East^(18)^. Also, there were other different reasons to select this province as the study area, including high traffic and commute, unfair distribution of income among people and high density (about 962 person/km^2^).

The online questionnaire was asked to fill out by either the head of the participated households or a member who is familiar with the status of household’s nutrition such as women^(18,23)^. An online standard questionnaire was used to collect the data because of several limitations of the government during this pandemic disease, including closing unnecessary jobs, social distancing, traffic ban, travel restrictions and isolation.

### Data collection and questionnaires, validity and reliability

The sample included 392 families residing in Tehran province which was determined using random sampling. Some questions in the questionnaire were duplicated from previous published manuscripts pertaining to previous virus outbreak in the world, including SARS, Ebola, HIV and Influenza^(18)^. Other items were also added to the final questionnaire according to COVID-19 condition. To access the goals of the study, a questionnaire composed of three sections was used. The first draft of the questionnaire was reviewed and revised by a group of twenty experts before starting the interview process. Experts included those from agricultural economics, psychology, environment and social science, and based on their opinion, the questionnaire was revised and, finally, confirmed^(18)^. The first section of the questionnaire included nine items to identify the status of food insecurity of Iranian households in the target area. The second section included thirty-seven items to measure household vulnerability status. Vulnerability included seven dimensions such as economic, social, cultural, human, physical, psychology and information. The final section focused on finding the factors underpinning households’ vulnerability. It was composed of seven items to evaluate economic factors, five items for cultural factors, seven items for social factors, five items for human factors, five items for physical factors, five items for psychological factors, four items for information factors and nine items for food security.

The benefits of this study were described on the first page of the online questionnaire: first, informing governmental administrations and global organisations about the status of households’ food security during COVID-19; second, contributing to policymakers to take quick action to improve the situation. As well as, Cronbach’s *α* coefficient, ranging from 0·7 to 0·9, was applied to assess the reliability of the research instruments.

### Assessment of food insecurity

A modified and validated version of the Household Food Insecurity Accecc Scale was used to assess and monitor the status of food security^(24)^. This instrument consists of a nine-item scale which happens during the past 4 week as a reference period^(5,25)^. Using this tool, respondents were requested to answer to each question with different experience including never, rarely, sometimes or often. Finally, food security index can be generated with a value range of 0–27 using their responses^(5)^. Based on this index, twenty-seven which is the higher value indicates the higher level of food insecurity of household. Then, food insecurity level can de divided into three categories including marginal, moderate and severe. Also, the Household Food Insecurity Access Scale is used to categorise the nutrition status of households in three levels including uncertainty about the food supply, inadequate quality and quantity of food.

### Measurement model

To determine the factors associated with food insecurity during COVID-19, the first-order structural equation modelling was applied. The structural equation modelling is a hypothesis testing method that states whether the indicators selected to measure latent variables are accurate^(26)^. Latent variables cannot be measured directly and must be figured out through observable variables that are directly measurable^(27)^. Therefore, to check the accuracy of the measurement model, the factors affecting food insecurity in COVID-19 condition were examined in three stages: (1) model reliability and validity; (2) unidimensionality; and (3) diagnostic analysis (13). Reliability by combined reliability and average extracted variance is a measure of the internal fit of the model and is estimated by the extent of the consistency of items selected for the measurement of a factor. In fact, it demonstrates the correlation of the items of the questionnaire with the related factor. If its value is higher than the recommended value, the items of the questionnaire are reliable and sufficiently valid to operationalise the model. In other words, it measures the target characteristic correctly and accurately.

Also, the standardised factor loadings for all indicators selected for the model constructs should be >0·5 and statistically significant. This provides adequate evidence to support the unidimensionality of the indicators selected for the measurement models. Hence, it can be claimed that the selected indicators were correctly chosen for the measurement of the research constructs. This shows that the respondents have had the same perception of the questions because different perceptions will reduce the measurement accuracy of the research instrument.

In structural equation modelling, when a satisfactory measurement model is attained (using confirmatory factor analysis), the structural model can be tested. To test the hypotheses, a path analysis method (evaluating the structural model) was used.

## Results

### Descriptive characteristics

Descriptive analysis of the study sample is demonstrated in Table 1. The range of the age of the participated households was between 25 and 89 years (mean = 48·2, sd = 12·26), and the average size of the targeted households was 3·69 (sd = 1·47). About 59·4 % of the respondents (*n* 233) were working full time. Almost 53·8 % of the participated households had determined personal savings, and 30·2 % of the households in Tehran province rented their home. About 29·8 % of mothers were employed outside the home, and 57·6 % of them were housewives in interviewed households. Almost 43·8 % of head of households elucidated that they have an adequate knowledge of the basics of nutrition. Finally, the average number of disease, literate and employed members in the final sample was 1·04, 1·03 and 0·92, respectively.

**Table 1:** Descriptive demographic and socio-economic characteristics of Iranian households in Tehran province

Variable	Statistical outputs for continuous factors			
	Minimum	Maximum	Mean	sd
I. Continuous				
(a) Age of head of household (years)	25	89	48·2	12·26
(b) Size of household	1	10	3·69	1·47
(c) Number of employed members	0	6	1·61	0·92
(d) Number of educated members	0	6	0·99	1·03
(e) Number of disease members	0	8	0·63	1·04
(f) Number of male children	0	5	0·36	0·69
(g) Number of female children	0	5	0·43	0·68

	Statistical outputs for categorical factors			
	Category	Frequency	Percentage	Mode
II. Categorical				
(a) Household head occupation status	1–3			
Employed		233	59·4	☑
Unemployed		63	16·0	
Retired		96	24·4	
(b) Household income	1–5			
Quintile (1)		96	24·4	
Quintile (2)		136	34·7	☑
Quintile (3)		91	23·2	
Quintile (4)		43	10·9	
Quintile (5)		26	6·6	
(c) Personal saving	1–2			
Yes		211	53·8	☑
No		181	46·1	
(d) Mother’s occupation status	1–3			
Employed		117	29·8	
Housewife		226	57·6	☑
Retired		49	12·5	
(e) Household head education status	1–4			
Illiterate		32	8·1	
Under diploma		78	19·8	
Diploma		85	21·6	
University		197	50·2	☑
(f) Residential status	1–3			
Rental		118	30·2	
Owner		226	57·6	☑
Other		48	12·2	
(g) Gender of head	1–2			
Male		313	79·8	☑
Female		79	20·1	
(h) Nutrition knowledge	1–5			
Very low		25	6·3	☑
Low		38	9·6	
Medium		172	43·8	
High		115	29·3	
Very high		42	10·7	

### Food insecurity

Table 2 displays the summary results of the Household Food Insecurity Access Scale questionnaire during the COVID-19 outbreak in Tehran province. About 2·8 % of total sample often experienced anxiety and uncertainty, while 57·6 % of those never had this situation. About 39 % of households had food security during the COVID-19 outbreak, while 61 % faced food insecurity. Also, about 25 %, 35·2 % and 34·9 % of the households faced marginal, moderate and sever food insecurity, respectively, during as the pandemic continued.

**Table 2:** The responses status of Iranian households to Household Food Insecurity Access Scale (HFIAS) questionnaire in Tehran province during COVID-19 outbreak

Level	Question	During COVID-19 outbreak				
		Never	Rarely	Sometimes	Often	Mean
Anxiety and uncertainty	(Q1) Worry about food	57·6	20·8	11·6	2·8	0·7
Insufficient quality	(Q2) Unable to eat preferred foods	49·0	26·6	15·0	9·2	0·9
	(Q3) Eat a limited variety of foods	50·0	24·3	15·7	9·9	0·8
	(Q4) Eat foods that you did not want to eat	54·9	22·9	12·6	8·5	0·7
Insufficient food intake	(Q5) Eat a smaller meal	57·2	22·9	11·9	8·8	0·6
	(Q6) Eat fewer meals in a day	70·3	15·7	8·8	5·1	0·5
	(Q7) No food to eat of any kind in the household	81·2	9·2	4·4	5·0	0·3
	(Q8) Go to sleep at night hungry	81·6	8·1	5·1	5·0	0·3
	(Q9) Go a whole day and night without eating	82·0	8·5	3·7	5·7	0·2

### Structural equation modelling

#### The fit indices of the model

The combined reliability of all the structures in the planned research model was more than 0·60, and their Cronbach’s *α* coefficient was higher than 0·70; in addition, the average extracted variance for all structures of the proposed research model was more than 0·50. Therefore, all latent variables of the proposed research model had good reliability and validity (Table 1). The standardised factor loading value (λ) of all selected indicators for the structures in question (above 0·5) was statistically significant (*P* < 0·01). Therefore, it can be stated that the selected indicators for measuring research structures have been chosen correctly (Table 3). The results showed that, in general, the mean of the extracted variance for the research structures (0·84 < average extracted variance < 0·98) was higher than the correlation between them (0·32 < *r* < 0·55). This result demonstrated that the diagnostic validity of the structural model was confirmed (Table 4).

**Table 3:** The results of fit of measurement models

Constructs	Measurement item	Reliability and validity statistics
Economic	(ECO1) Increase in the price of foods	AVE: 0·520 CR: 0·835 *α*: 0·774
	(ECO2) Decrease in the income and purchasing power	
	(ECO3) Become unemployed	
	(ECO4) The loss of sources of additional revenue	
	(ECO5) Increase the household’s expenditure	
	(ECO6) Sanction	
	(ECO7) Increase in exchange rate	
Cultural	(CU1) Weak educational services and closure of educational centres	AVE: 0·582 CR: 0·847 *α*: 0·761
	(CU2) Lack of inter-sectoral cooperation during the Corona era	
	(CU3) Unnecessary travel of some people	
	(CU4) Forgetting traditions and customs	
Social	(SO1) Existence of social insecurity	AVE: 0·534 CR: 0·842 *α*: 0·785
	(SO2) Existence of incorrect values and norms	
	(SO3) Low social trust	
	(SO4) Low public confidence in the government	
	(SO5) Trust foreign news sources	
	(SO6) Lack of powerful non-governmental organisations	
	(SO7) Lack of planning in the household	
Human	(HU1) Lack of access to medical staff	AVE: 0·679 CR: 0·872 *α*: 0·818
	(HU2) Low level of knowledge in sanitation	
	(HU3) High level of depression during quarantine	
	(HU4) Lack of sufficient knowledge of people in controlling COVID-19	
	(HU5) Lack of familiarity with the principles of proper nutrition	
Physical	(PH1) Lack of medical equipment	AVE: 0·651 CR: 0·859 *α*: 0·799
	(PH2) Lack of pharmaceutical items	
	(PH3) Weak infrastructure	
	(PH4) Expensiveness and lack of disinfectants and sanitary detergents	
	(PH5) Lack of required sanitary items (masks, gloves, all kinds of washing gels)	
Psychology	(PS1) Anxiety caused by illness	AVE: 0·637 CR: 0·853 *α*: 0·787
	(PS2) Sick of other family members	
	(PS3) Disappointing news on social media	
	(PS4) The existence of family tensions during the quarantine period	
	(PS5) Lack of exercise programmes in life	
Information	(IN1) Lack of timely notification	AVE: 0·659 CR: 0·835 *α*: 0·738
	(IN2) Low-speed Internet	
	(IN3) Lack of reliable sources of information on the control and treatment of COVID-19 outbreak	
	(IN4) Weak psychological counselling services	
Food insecurity	(FI1) Worried about not having enough food due to financial inability	AVE: 0·611 CR: 0·903 *α*: 0·879
	(FI2) Do not eat your favourite food because of your financial inability	
	(FI3) Restriction of food consumption (food diversity) due to financial inability	
	(FI4) Having to eat foods you don’t really like because of your financial inability	
	(FI5) Forced to eat less food due to financial inability	
	(FI6) Decreased number of meals (three meals) due to financial inability	
	(FI7) Having no food to eat at home due to financial inability	
	(FI8) Hungry night sleeping because of financial incapacity	
	(FI9) Spending a day without food due to financial inability	

AVE, average extracted variance; CR, combined reliability.

Suggested value: SRMR < 0·1 D-G1 > 0·05 D-G2 > 0·05 NFI > 0·90 RMS-Ɵ ≤ 0·12.

Estimated value: SRMR = 0·08 D-G1 = 0·624 D-G2 = 0 598 NFI = 0·99 RMS-*Ɵ* = 0·06.

**Table 4:** Correlations with square roots of the average extracted variance (AVE)

Constructs	1	2	3	4	5	6	7	8
1-ECO	0·84*							
2-CU	0·42†	0·93*						
3-SO	0·39†	0·38†	0·92*					
4-HU	0·45†	0·44†	0·47†	0·78*				
5-PH	0·36†	0·45†	0·44†	0·39†	0·82*			
6-PS	0·37†	0·46†	0·38†	0·44†	0·33†	0·91*		
7-IN	0·51†	0·32†	0·47†	0·40†	0·44†	0·52†	0·98*	
8-FI	0·42†	0·38†	0·44†	0·41†	0·48†	0·55†	0·41†	0·88*

* The square roots of AVE estimate.

† Correlation is significant at the <0·01 level.

### Measurement and structural model

To evaluate the fit of the structural model of the research with economic, cultural, social, human, physical, psychological, information and food insecurity variables, the indicators of goodness of fit were used (Table 3). The results indicated that the structural model of the research was appropriate and the research data provided good support for the theoretical model of the research.

**Fig. 1:**
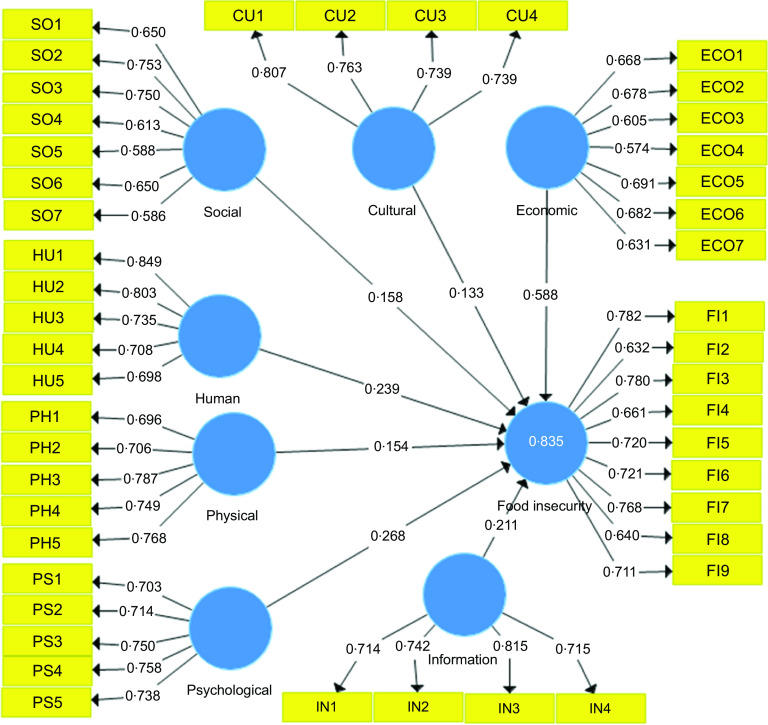
Structural equation modelling on determinants of household vulnerability to food insecurity during COVID lockdown in a mid-term period in Iran (Path model with standardised factor loadings)

**Fig. 2:**
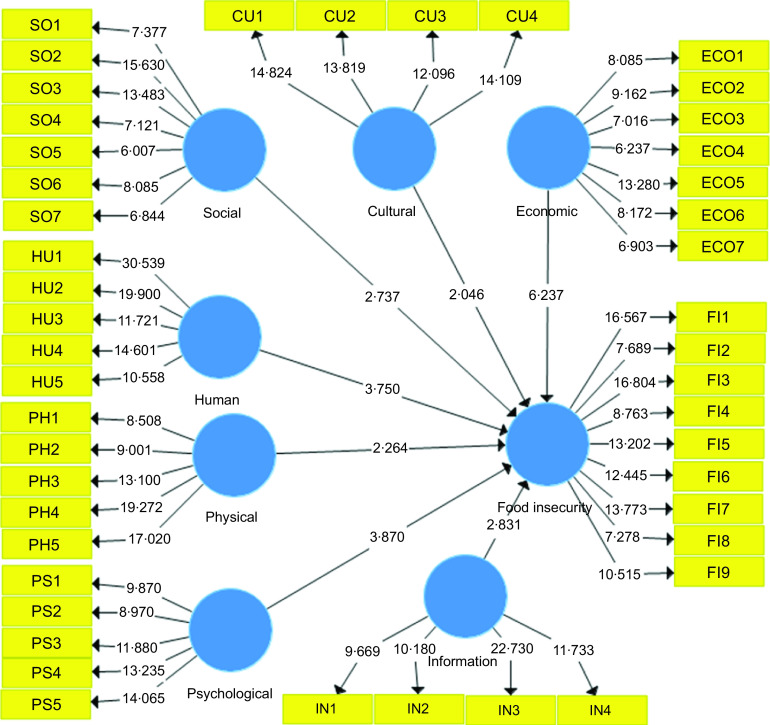
Structural equation modelling on determinants of household vulnerability to food insecurity during COVID lockdown in a mid-term period in Iran (Path model with *t*-values)

Figure 1 shows the correlation of each question with different vulnerability factors including social, cultural, economic, human, physical, psychological and information using standardised factor loadings. Figure 2 also explains all associations in path model using *t* value. Finally, the association of vulnerability factors with food insecurity of households during COVID-19 condition was demonstrated at the end of the structural model. Increasing households’ expenditure, international sanctions imposed on Iran by the USA, and decreasing in household purchasing power had the highest correlation with economic factors as the first dimension of households’ vulnerability during COVID-19 outbreak. Weak educational services and lack of inter-sectoral cooperation during this pandemic disease had the greatest correlation with social factor of household vulnerability. Social and cultural dimensions included existence of incorrect values and norms among Iranian households in the target area; low social trust and lack of powerful Non-Governmental Organisations were identified as important factors affecting household vulnerability at the mid-term period of the COVID-19 outbreak. Lack of access to medical staff and low level of knowledge of sanitation were found to be indispensable factors in human aspect of household vulnerability to food insecurity during COVID-19 outbreak. Considering physical aspect, weak infrastructure and lack of required sanitary items including masks, gloves and all kinds of washing gels were associated with household vulnerability. Finally, lack of reliable sources of information on the control and treatment of COVID-19 disease and low-speed internet had the higher correlation with information aspect of households’ vulnerability during COVID-19 pandemic disease. As Fig. 2 shows that the *t* value of all factors in path model was significant. Therefore, the interpretation of these factors is reliable and accurate. Also, the final effects of the variables on the behaviours of Iranian households in dealing with food insecurity in the study area are presented in Figs. 1 and 2. The bootstrapping method was used to test the research hypotheses. All the research hypotheses were confirmed. The variables were able to explain 83·5 % of food insecurity within the sample. As Fig. 1 demonstrates, latent variables including economic, physiological and human aspects of household vulnerability have the greatest effect on food insecurity during COVID-19 outbreak. On the other hand, cultural and physical aspects of the vulnerability had the lowest effect on food insecurity at the onset of the pandemic.

## Discussion

Due to growing concern about food insecurity status during the COVID-19 pandemic, many national and international institutions and authorities are mounting special efforts to assess and monitor food security status and the different dimension of households’ vulnerability around the world. The results of the present study revealed that 61 % of the participated households faced marginal, moderate and severe food insecurity. About 34·9 % of them had severe food insecurity. A study showed that, in the earliest and short-term stage of this pandemic disease, about 57 % of Iranian households in this province faced food insecurity^(18)^. This comparison confirms the increase in food insecurity level of Iranian households in Tehran province in a mid-term period of COVID-19 outbreak.

According to the result, arduous economic conditions were identified as important contributors to prevailing household food insecurity during COVID-19 outbreak. Most businesses are closed in Tehran province as a high risk zone of prevalence of COVID-19 virus in Iran, and people face economically formidable challenges in this province. Due to the decrease in production, the price of goods, especially foods and beverage, soared and the purchasing power of people declined in Iran, while household expenditure increased. As in other developing countries, the value of local currency decreased dramatically. Finally, most households could not prepare their needed food ingredients. It is predictable that many developing economies will face arduous economic challenges, including loss of tourism and trade, become weak of health care systems, dwindling remittances, reduction of capital flows and increase in domestic and foreign debts. An important aspect of this viewpoint is the human and economic sectors that the global recession will have on parent industries and core markets, which account for approximately one-third of GDP and about 70 % of total employment in emerging and developing markets. Also, according to the participants’ response, the imposition of unilateral and plurilateral sanctions of USA on the country increases the vulnerability of households to food insecurity during COVID-19 outbreak. Sectoral sanctions created several serious limitations in accessing to indispensable medical equipment and medicines, including protective and needed equipment for healthcare staffs, ventilator, oxygen generator and respirators. Also, these sanctions weaken the government to import the needed and related health equipment including gun and mask to struggle with this pandemic disease. The destructive effect of such sanctions is complicated in Iran, especially during the COVID-19 pandemic, impacting almost all human rights, particularly lifestyle. This means that going-on these situations is simply a crime. Some studies contended that economic factors should be considered as an important factor in people’s vulnerability to food insecurity in the world^(5,23,28–30)^.

Inappropriate psychological conditions which were identified as the second important factor in fitted model can increase the households’ vulnerability to food insecurity during COVID-19 outbreak. Psychological distress, depression, anxiety and stress experienced by people can weaken their potential to struggle with this pandemic disease in long-term period. Although some studies showed that anxiety and depression did not significantly differ among some communities in Iran (among Iranian medical students as the case study) in the early stages of the COVID-19 pandemic^(31,32)^, but our results demonstrated that this negative effect of inappropriate psychological aspect may increase households’ vulnerability with the passing of time as other contended^(33)^. The existence of families’ tensions during the quarantine period was determined as a most important factor of mental health and psychological aspect in increasing the households’ vulnerability to food insecurity during COVID-19 outbreak. As the disease grows, authorities are asking people to stay at home and, therefore, many people have to spend extended time with their roommates or families. This situation can ultimate conflict, whether it be full-blown argumentations or passive-aggressive comments. Clear and honest communication can be helpful to cease this conflict during COVID-19 quarantine. In the following, disappointing news on social media was identified as another important psychological factor in increasing household vulnerability to food insecurity during this pandemic. Iranian people hear frustrating news every day, like other people around the world, about the number of deaths caused by this disease (about 200 cases each day in the country) in social media, TV and radio which caused serious mental problems for them including fear, apprehension, anxiety and despair. However, this crisis highlights the particular strengths of social media in spreading important health care and medical training to combat this pandemic disease.

Human aspects of vulnerability were identified as the next important factor in the increase in food insecurity level during COVID-19 outbreak. Lack of access to medical staff and health care workers and a low level of people’s knowledge of sanitation were important in the increase in household vulnerability. Health care staffs are in the frontline of battle with COVID-19, and access to them is inevitable and undeniable to control this pandemic, but according to WHO’s report, there is one doctor per 2000 people in Iran, which is ranked 130th in the world. It should be noted that Italy has one medical staff per 169 people, which is the highest number in the world. According to state media, about 100 000 nurses and 15 000 medical staff are activing in the battle with this pandemic disease in Iran and a number of them are losing their lives every day. The results showed that insufficient information about COVID-19 or insufficient access to the resources which would allow people to maintain the hygiene and sanitation standards required for effective protection is the next important factor in human aspect of vulnerability to food insecurity. As this is a new disease worldwide, Iranian people did not have the necessary training to deal with such a disease. Given the coronavirus (COVID-19) pandemic, washing hands frequently with water and soap is one of the easiest, cheapest and most inevitable ways to prevent the spread of the virus.

Lack of sufficient information makes Iranian households vulnerable to food insecurity during COVID-19 outbreak. The people receive different information on how to combat COVID-19 from different sources, which makes them confused. Distinct ways to control the spread of this virus which are recommended by different experts and institutions, dissimilar news about the discovery of vaccine around the world, and different statistics of death from distinct information resources are a summary of information contradictions.

Finally, although other aspects including social, physical and cultural factors were ranked in the next, people and authorities should consider their importance to control the spread of the COVID-19. Also, we believe that the rank of these aspects can be distinct in different places and countries around the world due to variations in geography and culture.

## Conclusion and policy implications

As the COVID-19 outbreak continues around the world, other threats to human health emerge, including access to food. Our analysis of predictors of vulnerability to food insecurity addressed multiple aspects of that vulnerability, including economic, human, cultural, social, physical, psychological and information. We found that all these aspects significantly affect households’ food in/security during COVID-19 outbreak. For developing countries such as Iran, which confront serious structural vulnerabilities, strengthening public health systems, addressing and monitoring the problems posed by informality and implementing health reforms supporting sustainable and strong growth, once a health crisis occurs, can helpful to reduce the vulnerability to food insecurity.

From a policy perspective, our results suggest that authorities and policymakers must provide economic and financial support to vulnerable households. Such support should include direct payments, free food packages, postponement of bank debts, guaranteed income supplement, loan guarantee for small- and medium-sized enterprises and increasing credit availability. While these economic strategies can enhance people’s ability to cope with vulnerability to food insecurity during COVID-19, international sanctions can make achieving these solutions very difficult or even impossible. Broad sectoral sanctions against Iran should urgently be re-evaluated in light of their debilitating impact on the health and human rights of ordinary people. USA has imposed severe, arduous sanctions on Iran harming millions of innocent people. Before it is too late to avert a great human catastrophe, these international actors should provide transparent information, send necessary humanitarian assistance and lift economic and financial sanctions until the disease is completely gone.

To decrease family tensions during the quarantine period, providing free psychological and behavioural programmes on TV and social media are recommended. Broadcasting uplifting and varied programmes on TV as well as limiting the broadcast of sad and disappointing news are other suggested strategies by social psychologists globally and in Iran to improve human aspect of households’ vulnerability. Also, and in line with the COVID-19 response and preparedness, a broader approach including hygiene is needed. Approaches, principles and learnings from the market-based sanitation need to be adapted to the broader market impact for local and national hygiene markets, especially in the context of COVID-19 and post-COVID-19, with a focus on public health.

The results of the present study can be applied by authorities and policymakers in similar countries to prioritise which aspects of households’ and individuals’ vulnerability can be addressed by government actions. Future studies should try to determine the factors affecting households’ food insecurity, especially among vulnerable groups such as refugees and asylum seekers in their countries because the rank of vulnerability aspects can be distinct according to geographical disparities.
