# Determinants of household vulnerability to food insecurity during COVID-19 lockdown in a mid-term period in Iran – ERRATUM

**DOI:** 10.1017/S1368980021000872

**Published:** 2021-05

**Authors:** Mohammad Reza Pakravan-Charvadeh, Moselm Savari, Haider A Khan, Saeid Gholamrezai, Cornelia Flora

During production of the above-mentioned article, Mohammad Reza Pakravan-Charvadeh was inadvertently attributed with all four author affiliations. The only correct one is Lorestan University.

This has since been corrected. Cambridge University Press apologise for this oversight.
